# MIMO and PDM-based intersatellite optical link for high-speed data transfer and remote sensing application

**DOI:** 10.1371/journal.pone.0313342

**Published:** 2024-11-12

**Authors:** Sushank Chaudhary, Yahui Meng, Abhishek Sharma, Muhammad Ali Naeem

**Affiliations:** 1 School of Computer, Guangdong University of Petrochemical Technology, Maoming, China; 2 School of Science, Guangdong University of Petrochemical Technology, Maoming, China; 3 Department of Electronics and Communication Engineering, National Institute of Technology, Hamirpur, India; Parul University, INDIA

## Abstract

Inter-Satellite Optical Wireless Communication (Is-OWC) is a pivotal technology for advancing global connectivity and the effectiveness of space-based operations. It serves as the linchpin for various applications such as global internet coverage, Earth observation, and remote sensing, bolstering our capacity to monitor the planet and deliver essential services. This paper presents the design and performance evaluation of a Polarization Division Multiplexing (PDM) and Multiple Input Multiple Output (MIMO)-based Is-OWC system operating at a data rate of 60 Gbps. The system was tested under varying transmission distances, atmospheric turbulence, and pointing error conditions. At a transmission distance of 10,000 km, the system achieved a Bit Error Rate (BER) of 6.76 × 10⁻^3^ for Channel 1 (X polarization) and 7.1 × 10⁻^3^ for Channel 4 (Y polarization), both within Forward Error Correction (FEC) limits. The introduction of a 4 × 4 MIMO configuration extended the transmission range to 14,000 km, with a corresponding BER of 9.1 × 10⁻^3^ for Channel 1 and 8.7 × 10⁻⁵ for Channel 4. Eye diagrams confirm successful signal reception, demonstrating that the system can maintain high data rates and low error rates over long distances. The proposed PDM-MIMO system showcases high-capacity, robust performance under challenging conditions, such as turbulence and pointing errors, validating its suitability for space-based communication applications. These findings highlight the potential of the system for future deployments in satellite networks, offering reliable, high-throughput, and low-latency data transmission over extended distances.

## 1. Introduction

Satellite communication has long been a cornerstone technology in our quest for seamless global connectivity, with an ever-growing constellation of satellites orbiting the Earth [1]. The ability to transmit data across vast expanses of space has opened the door to a myriad of applications, ranging from global internet coverage to Earth observation and beyond. In this interconnected age, the role of inter-satellite communication has gained paramount significance, calling for innovations that can enhance the efficiency and scope of space-based operations [2, 3]. One key challenge in the realm of satellite communication is the need for high-capacity, low-latency data transfer between satellites [4, 5]. This challenge becomes even more pronounced when considering the critical applications of remote sensing, which require the reliable and rapid transmission of vast amounts of data from satellites to Earth’s surface.

**Fig 1:**
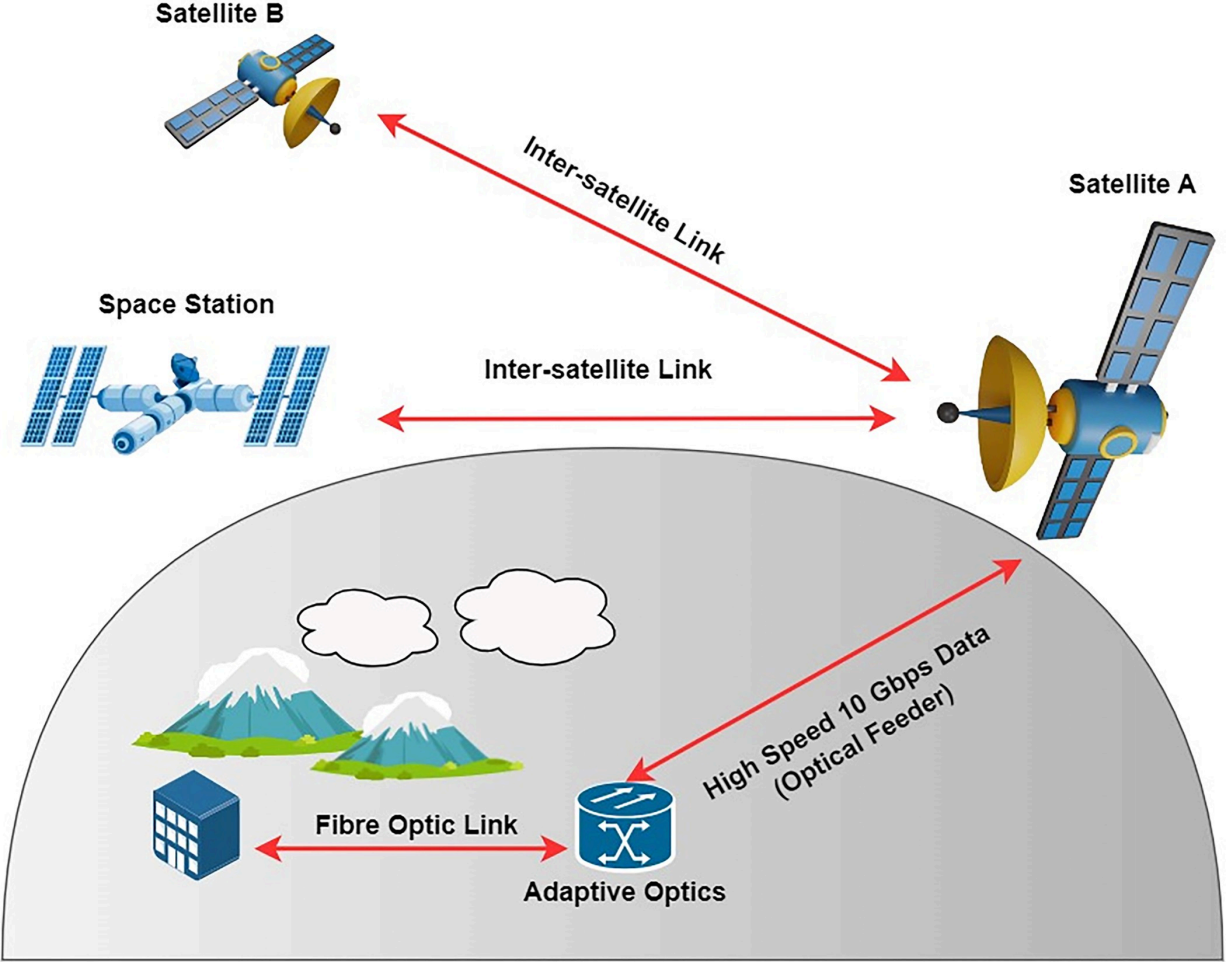
Intersatellite Optical Wireless Communication (Is-OWC) scenario.

Fig 1 demonstrates the inter-satellite communication system (Is-OWC) implementation scenario. As we endeavor to monitor our planet and provide essential services such as weather forecasting, disaster management, and environmental monitoring, the demand for advanced inter-satellite communication solutions becomes increasingly apparent [6]. In response to this demand, our research focuses on the development of an economically efficient Is-OWC, which stands as a pivotal enabler for high-speed data transfer between satellites. This technology not only strengthens our existing satellite networks but also opens up new horizons in the realm of Earth observation and remote sensing [7, 8]. In this work, we propose a cutting-edge approach that combines multiple input multiple output (MIMO) technology with a polarization division multiplexing scheme (PDM), creating a system capable of supporting multiple high-capacity data channels over challenging distances.Is-OWC systems are notably advantageous, boasting large channel capacity, secure data transmission, high-speed links, resistance to electromagnetic and RF interference, low power requirements, cost-effectiveness, freedom from spectrum licensing, low beam divergence, minimal security upgrades, and rapid deployment [9, 10]. However, Is-OWC systems grapple with several challenges, including background noise interference, satellite vibration, and transmitter-receiver misalignment [11–15]. Numerous modulation strategies have been explored to address these challenges, with the selection of the optimal modulation technique serving as a crucial determinant of system performance. The core of our proposed Is-OWC technology lies in the synergy of two advanced communication techniques: PDM and MIMO. PDM leverages the orthogonal polarization states of light to multiplex multiple data streams onto a single optical carrier, effectively increasing the data-carrying capacity of the communication channel [16–18]. By exploiting these polarization states, we can transmit and receive multiple independent data streams simultaneously, enabling a substantial increase in data throughput. On the other hand, MIMO technology [19–23] leverages multiple antennas at both the transmitter and receiver to create spatial diversity, allowing for the transmission of multiple parallel data streams that can be independently processed. In our study, we integrate these two powerful techniques, employing PDM to enable six channels, each carrying 10 Gbps of non-return to zero encoded data, while MIMO technology enhances the robustness and reliability of the communication link, thus ensuring that high-capacity data transfer can occur over challenging distances. This combined approach not only significantly boosts the overall data throughput but also fortifies the communication system against impairments induced by space turbulences, making it a promising solution for next-generation inter-satellite communication. The selection of PDM-MIMO technology addresses the need for high-capacity communication with resilience against signal fading and interference. This configuration enables spectral efficiency and spatial diversity, ensuring reliable communication in satellite networks under varying condition. This research offers several significant contributions to the field of inter-satellite communication and its applications, particularly in the context of remote sensing:

- We introduce an economically efficient Is-OWC that combines PDM and MIMO techniques to enable high-capacity data transfer between satellites.
- We provide a comprehensive evaluation of the Is-OWC system’s performance over challenging distances, including a demanding 14,000 km link, subjected to space turbulences and transmission errors, demonstrating its robustness and reliability.
- Our work enhances Earth observation and remote sensing capabilities by enabling the rapid and reliable transmission of vast amounts of data from satellites to Earth’s surface, thereby advancing our ability to monitor and collect crucial information about our planet.
- Ultimately, this research contributes to the advancement of inter-satellite communication technology, with a specific emphasis on applications that demand high-capacity data transfer, including remote sensing. These findings collectively demonstrate the innovative and practical aspects of our research, underscoring its significance in the field of satellite communication and its potential to drive advancements in remote sensing and related domains.

The remaining section of the manuscript can be divided as follows: Section 2 presents the related work to Is-OWC links, Section 3 presents the Channel modeling, Section 4 presents the Proposed RGB-PDM-Is-OWC Link, Section 5 presents the results and discussions,Section 6 presents the Conclusion and Section 7 presents the Future scope.

## 2 Related work

This section is dedicated to examining research and developments related to the Is-OWC scheme. Researchers in 2021 [24] highlights the importance of free-space optical (FSO) communication for Is-OWC systems, enabling efficient satellite communication over long distances. The study establishes a 45,000 km Is-OWC link with a target low error rate and introduces advanced techniques like MIMO and wavelength division multiplexing (WDM) for improved performance. The authors in 2022 [25] investigate Mode Division Multiplexing (MDM) for advanced optical wireless networks, addressing issues of inter-channel interference and modes coupling. They introduce an interference reduction technique using transmitter diversity (TD) in a 2λ × 2HG modes × 10 Gbps Is-OWC system, comparing TD methods (NRZ-return to zero and NRZ-alternate mark inversion) with non-TD (NRZ–NRZ) over a 5000 km Is-OWC distance. Utilizing Hermite Gaussian (HG) modes (HG00 and HG01) at different wavelengths yields a total capacity of 40 Gbps. Their results reveal improved Is-OWC system performance with the NRZ-AMI arrangement, surpassing the non-TD NRZ–NRZ system in terms of Bit Error Rate (BER). Another work [26] focuses on improving the reach of Is-OWC systems to meet the growing demand for internet connectivity. It introduces a hybrid system that combines Optical Code Division Multiplexing (OCDMA) and MDM to achieve a 120 Gbps capacity. The study evaluates different optical amplifiers, including semiconductor optical amplifiers (SOA), erbium-doped fiber amplifiers (EDFA), and Raman fiber amplifiers (RFA). It also explores the combined effects of Laguerre–Gaussian (LG) and Hermite–Gaussian (HG) modes with these amplifiers. The results indicate that the proposed system can cover a 5000 km Is-OWC link with acceptableBER, with EDFA using LG modes being the most effective approach. In 2023 [27] authors introduced a high-capacity Is-OWC system with a capacity of 3.84 Tbps. This system employs WDM and PDM to transmit three independent 40 Gbps data streams using 3-D orthogonal modulation techniques. Comparing two modulation methods, it is found that the proposed MDRZ–DQPSK–PolSK system achieves a superior Is-OWC distance of 65,000 km compared to CSRZ–DQPSK–PolSK, which reaches 58,000 km. This improvement is achieved while maintaining acceptableBER and Q factor levels. Another study [28] presents a point-to-point (P2P) superdense wavelength division multiplexing (SDWDM) system for radio over intersatellite optical wireless communication (Ro-IsOWC). The system features 96 channels, each with a 40 GB/s data rate and separated by a 100 GHz bandwidth. The study evaluates the system’s performance using advanced modulation schemes, including CSRZ, DRZ, and MDRZ, in clear weather conditions. It also analyzes internal parameters like transceiver aperture diameter and optical amplifier gain. The results show that CSRZ modulation performs well, achieving a BER of 10^−09^ with a 5.5 dB quality factor for all wavelengths. However, signal quality deteriorates beyond data rates of 40 GB/s and link distances of 10,000 km. A new study [11] addresses the growing need for high-speed data transmission in satellite communication, specifically through Inter Satellite Optical Wireless Communication (Is-OWC). To overcome challenges like limited transmission range, the research presents an 8-channel WDM system with Orthogonal Frequency Division Multiplexing (OFDM). Using a hybrid MDM and PDM approach, each channel starts at 320 Gbps but reaches 640 Gbps. Simulation results demonstrate a maximum data rate of 5.12 Tbps and a reach of 48,000 km with acceptable error rates, making this system a promising solution for high-speed satellite connectivity.Our present research is centred on the operation of Is-OWC links within the Medium Earth Orbit (MEO) range, spanning from 6000 to 13000 km above Earth’s surface. This specific orbital range holds immense promise for a range of critical applications, including high-speed data transfer, secure information exchange, and reliable connectivity among satellites and space-based platforms. By optimizing Is-OWC technology in this MEO region, we aim to advance global communication, navigation, and remote sensing capabilities, contributing to the ever-expanding frontiers of space-based solutions. In pursuit of this objective, we’ve established an Is-OWC link, empowered by PDM-MIMO technology, with the capacity to transmit data at a rate of 60 Gbps across a maximum link distance of 13,000 km. Furthermore, we’ve deliberately introduced space-related turbulences, including transmission and reception pointing errors, to assess the performance of the PDM-MIMO Is-OWC link. Our evaluation focuses on critical factors such as bit error rates and the characteristics of eye diagrams. The subsequent sections of this paper encompass the modelling of the proposed PDM-MIMO Is-OWC system (Section 3), presentation and discussion of modelling results (Section 4), and a comprehensive conclusion summarizing our findings (Section 5).

## 3. Channel modelling

This section presents the mathematical framework used to model the free space optical channel in the proposed PDM-MIMO-Is-OWC. The system’s performance is influenced by several factors, including free space path loss, pointing errors, and atmospheric turbulence. The following equations outline the modeling of received optical power, pointing error losses, and the impact of atmospheric turbulence, which together provide insights into the overall system performance.

### 3.1. Free space path loss model

The received optical signal power *P*_*R*_ at the receiver is given by [29–32]:

$$P_{R}=P_{T}\;\eta_{T}\;\eta_{R}\;{\left(\frac{\lambda }{4\;\pi \;Z}\right)}^{2}\;G_{T}\;G_{R}\;L_{T}\;L_{R}$$ (1)

Where, the transmitted and received optical powers are denoted as *P*_*T*_ and *P*_*R*_, respectively. Additionally, *η*_*T*_ and *η*_*R*_ represent the optical efficiencies of the transmitter and receiver, while *λ* stands for wavelength. *Z* represents the distance between the transmitter and receiver, *G*_*T*_and *G*_*R*_ denote the gains of the transmitting and receiving telescopes, and *L*_*T*_and *L*_*R*_represent the pointing loss factors for the transmitter and receiver. The term $\left(\frac{\lambda }{4\;\pi \;Z}\right)$ represents the free space path loss over the transmission distance *Z*. This equation provides the basis for calculating the received signal power over large inter-satellite distances. The gain of the transmitter and receiver telescopes *G*_*T*_ and *G*_*R*_ can be approximated as:

$$G_{T}={\left(\frac{\pi D_{T}}{\lambda }\right)}^{2}$$ (2)

$$andG_{R}={\left(\frac{\pi D_{R}}{\lambda }\right)}^{2}$$ (3)

where, *D*_*T*_ and *D*_*R*_corresponds to the diameter of the transmitters and receiver’s telescope, respectively.

### 3.2. Pointing loss modelling

Pointing errors due to misalignment between the transmitter and receiver can significantly impact system performance. The pointing loss factors *L*_*T*_ and *L*_*R*_ can be modelled as [29–31, 33]:

$$L_{T}={\left(-G_{T}\theta_{T}\right)}^{2}$$ (4)

$$and\;L_{R}={\left(-G_{R}\theta_{R}\right)}^{2}$$ (5)

where, the angle *θ*_*T*_ and *θ*_*R*_represents the azimuthal pointing error of the transmitter and receiver. These loss factors quantify the reduction in received power due to pointing errors, which are especially critical in long-range inter-satellite links.

### 3.3. Atmospheric turbulence model

Atmospheric turbulence causes random variations in the optical signal as it propagates through the medium. This effect is modeled using the Gamma-Gamma distribution to account for small and large-scale eddies, with the probability of a given intensity *I* at the receiver described by [29–31, 33]:

$$P(I)=\frac{2{\left(\alpha \beta \right)}^{\frac{\alpha +\beta }{2}}}{\Gamma (\alpha )\Gamma (\beta )}I^{\frac{\alpha +\beta }{2}-1}K_{\alpha -\beta }(2\sqrt{\alpha \beta I})$$ (6)

where α and β are parameters related to the variances of small and large-scale eddies; *K*_α−β_(.) is the modified Bessel function of the second kind Γ(.) is the Gamma function. The The values α and β are functions of the atmospheric turbulence strength and are approximated by the following expressions:

$$\alpha =\exp\left[\frac{0.49\sigma_{R}^{2}}{{\left(1+1.11\sigma_{R}^{12/5}\right)}^{7/6}}\right]-1$$ (7)

$$\beta =\exp\left[\frac{0.51\sigma_{R}^{2}}{{\left(1+0.69\sigma_{R}^{12/5}\right)}^{7/6}}\right]-1$$ (8)

where $\sigma_{R}^{2}$ is the Rytov variance, a parameter that quantifies the strength of atmospheric turbulence and is given by:

$$\sigma_{R}^{2}=1.23C_{n}^{2}k^{7/6}Z^{11/6}$$ (9)

Here, $C_{n}^{2}$ is the refractive index structure parameter, *k* is the optical wavenumber, and *Z* is the transmission distance.

### 3.4. BER estimation

To estimate the Bit Error Rate (BER) under Gaussian noise conditions, the following model is applied. Assuming Gaussian noise with standard deviations σ_0_ and σ_1_, the BER can be estimated using the formula [34]:

$$P_{e}=\frac{M}{N+M}P_{e0}+\frac{N}{N+M}P_{e1}$$ (10)

where *P*_0_ and *P*_1_ represent the probabilities of the transmitted symbols, *M* is the number of samples for logical ’0’, *N* is the number of samples for logical ’1’.The error probabilities *P*_*e*0_ and *P*_*e*1_ are calculated as:

$$P_{e0}=\frac{1}{2}\mathrm{erfc}\left(\frac{S-\mu_{0}}{\sqrt{2}\sigma_{0}}\right)$$ (11)

$$P_{e1}=\frac{1}{2}\mathrm{erfc}\left(\frac{\mu_{1}-S}{\sqrt{2}\sigma_{1}}\right)$$ (12)

where *μ*_0_ and *μ*_1_ are the average values of the sampled signals for logical ’0’ and ’1’, respectively, *σ*_0_ and *σ*_1_ represent the standard deviations of the noise for each symbol, *S* is the decision threshold for symbol detection. The complementary error function *erfc* is used to evaluate the probability of error.

## 4. Proposed RGB-PDM-Is-OWC link

**Fig 2:**
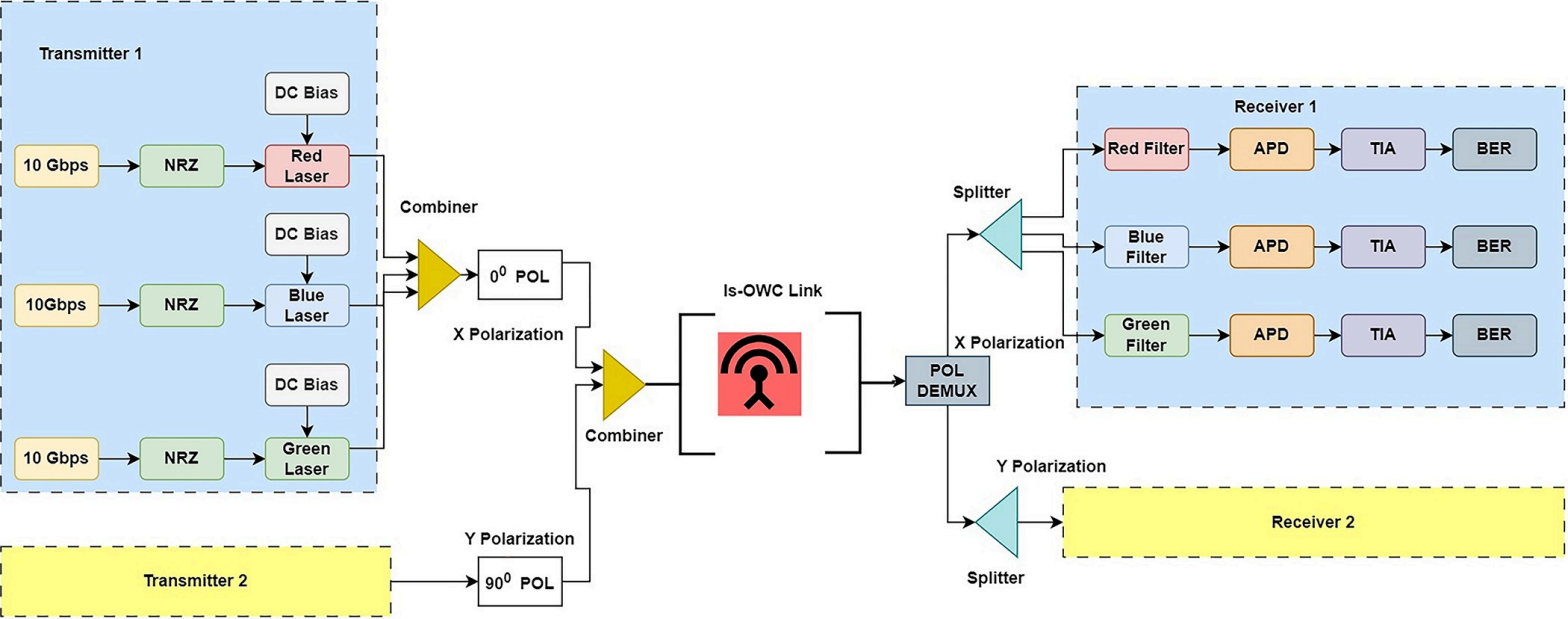
6 × 10 Gbps RGB-PDM-Is-OWC link.

In Fig 2, the schematic representation of PDM–MIMO-Is-OWC system is depicted, which is meticulously designed and modelled within the OptiSystem™ software.

OptiSystem™ was selected for its ability to simulate realistic environmental conditions such as turbulence and pointing errors, providing reliable insights before practical deployment. This illustration depicts the generation of the optical carrier, achieved through a continuous wave (CW) laser with a power level of 0 dBm. As shown, 3 lasers red, green and blue with the wavelength of 650 nm, 530 nm and 450 nm respectively are used in both the transmitters. For polarization, we employ states of polarization (SOP) set at 0° and 90°, thereby establishing the X polarization and Y polarization components for the optical carrier such that transmitter 1 carrying RGB laser is given X polarization while transmitter 2 carrying RGB laser is given Y polarization. Both polarization states are efficiently combined using a polarization division multiplexer, facilitating their transmission over the Is-OWC link. In the receiver section of our Is-OWC system, a polarization division de-multiplexer assumes a pivotal role in the precise separation of channels based on their unique polarization states. This component is instrumental in the effective reception of optical signals, allowing our system to distinguish between multiple channels transmitted with varying polarization orientations. The technology or method underlying our polarization division de-multiplexer offers robust solutions to the challenge of channel separation, thereby ensuring the preservation of data integrity and enabling the reception of high-capacity data streams. Moreover, we harness the power of a 4×4 MIMO configuration within our receiver system to enhance the overall signal reception process. By employing multiple antennas, this MIMO configuration significantly improves data transmission reliability, data rates, and interference mitigation. Our implementation of MIMO is detailed, emphasizing its substantial benefits in terms of increased data capacity and diversity, ultimately playing a vital role in the stellar performance of our Is-OWC system. Additionally, our receiver system incorporates optical filters to select specific wavelengths corresponding to desired signals. These filters operate within well-defined wavelength ranges, thus contributing to the selectivity and quality of the received signals. Subsequently, our system employs avalanche photo-diodes to transform optical signals into electrical signals, and a low-pass filter further processes these signals. The role of the low-pass filter is to attenuate high-frequency components while permitting the passage of the baseband signals, thus refining the overall signal quality. The quality assessment is conducted using a BER tester, an essential component for evaluating signal integrity and the overall performance of our Is-OWC system. This comprehensive receiver system enables precise reception and evaluation of high-capacity data streams, affirming the effectiveness of our approach in advanced inter-satellite communication for remote sensing applications and beyond. The Table 1 shows the other simulator parameters considered for modelling of proposed Is-OWC system.

**Table 1:** Simulation parameters.

Component name	Parameters	Value
Simulation Window	Sequence Length	1024 bits
	Samples per bit	64
Directly Modulated Laser	Wavelength	650 nm, 530 nm and 450 nm
	Power	10 dBm
	Linewidth	10 MHz
	Extension Ratio	10 dB
	Noise Bandwidth	1 THz
APD	Gain	3 dB
	Responsivity	1.2 A/W
	Ionization Ratio	0.9
	Dark Current	10 nA
	Thermal Noise	100e-024 W/Hz
Transimpedance Amplifier	Open loop voltage gain	600 Ohm
	Total Input Capacitance	3 pF
	Feedback Resistance	0.01e + 006 Ohm
	Noise Bandwidth	1 e + 009 Hz
	Absolute Temperature	298 K

## 5. Results and discussion

**Fig 3:**
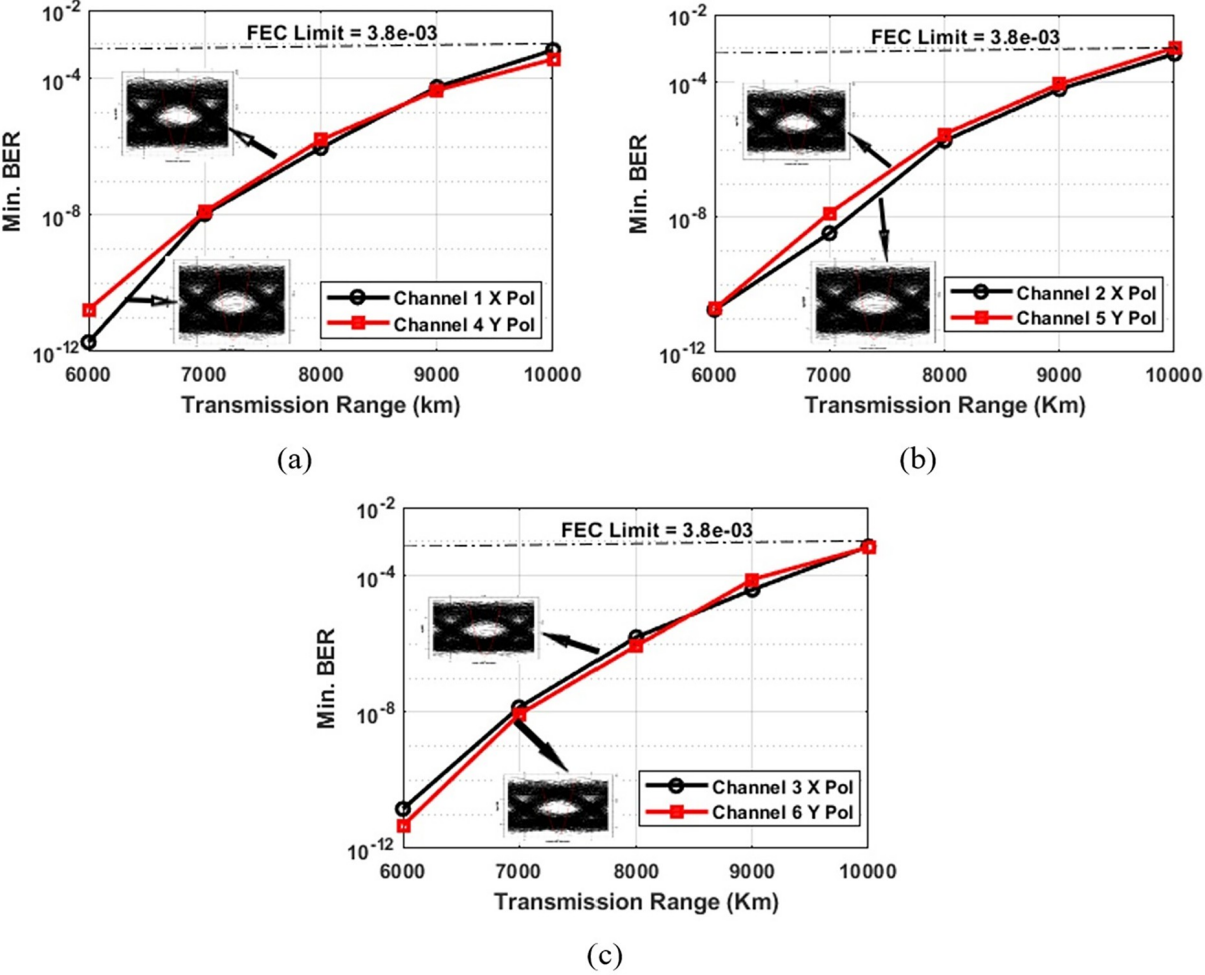
BER performance across varying transmission ranges in the PDM-Is-OWC system (without MIMO).

In this section, the outcomes of modeling the proposed 60 Gbps PDM-MIMO–Is-OWC system are presented and analyzed. Initially, the system was tested without MIMO or pointing errors, as shown in Fig 3. Fig 3(A) illustrates the recorded BER for channels 1 and 4 transmitted on the X and Y polarizations, utilizing the NRZ encoding format. Specifically, at a distance of 10,000 km within the Is-OWC link, the BER values for Channel 1 with X polarization stand at 6.76 × 10^-03^and 7.1 × 10^-03^for Channel 4 with Y polarization. These results comfortably fall within the Forward Error Correction (FEC) limit of 3.8 × 10^−03^. The clear eye openings in the inset show successful reception of the signal at a range of 9,000 km. Similarly, Fig 3(B) displays the Bit Error Rate (BER) results for channels 2 and 5, transmitted with X and Y polarizations, respectively, using the NRZ encoding format. At a distance of 10,000 km, Channel 2 with X polarization exhibits a BER of 6.87 × 10^−03^, while Channel 4 with Y polarization shows a BER of 3.9 × 10^−03^, both within the FEC limit. The inset highlights clear eye openings, indicating successful signal reception at a range of 9,000 km. Lastly, Fig 3(C) shows the BER for channels 3 and 6, transmitted on X and Y polarizations, utilizing NRZ encoding. At 10,000 km, Channel 3 with X polarization shows a BER of 7.19 × 10^−03^, and Channel 6 with Y polarization shows 6.81 × 10^−03^. These results fall within the acceptable FEC limit, and the eye openings at 9,000 km indicate successful signal reception.

**Fig 4:**
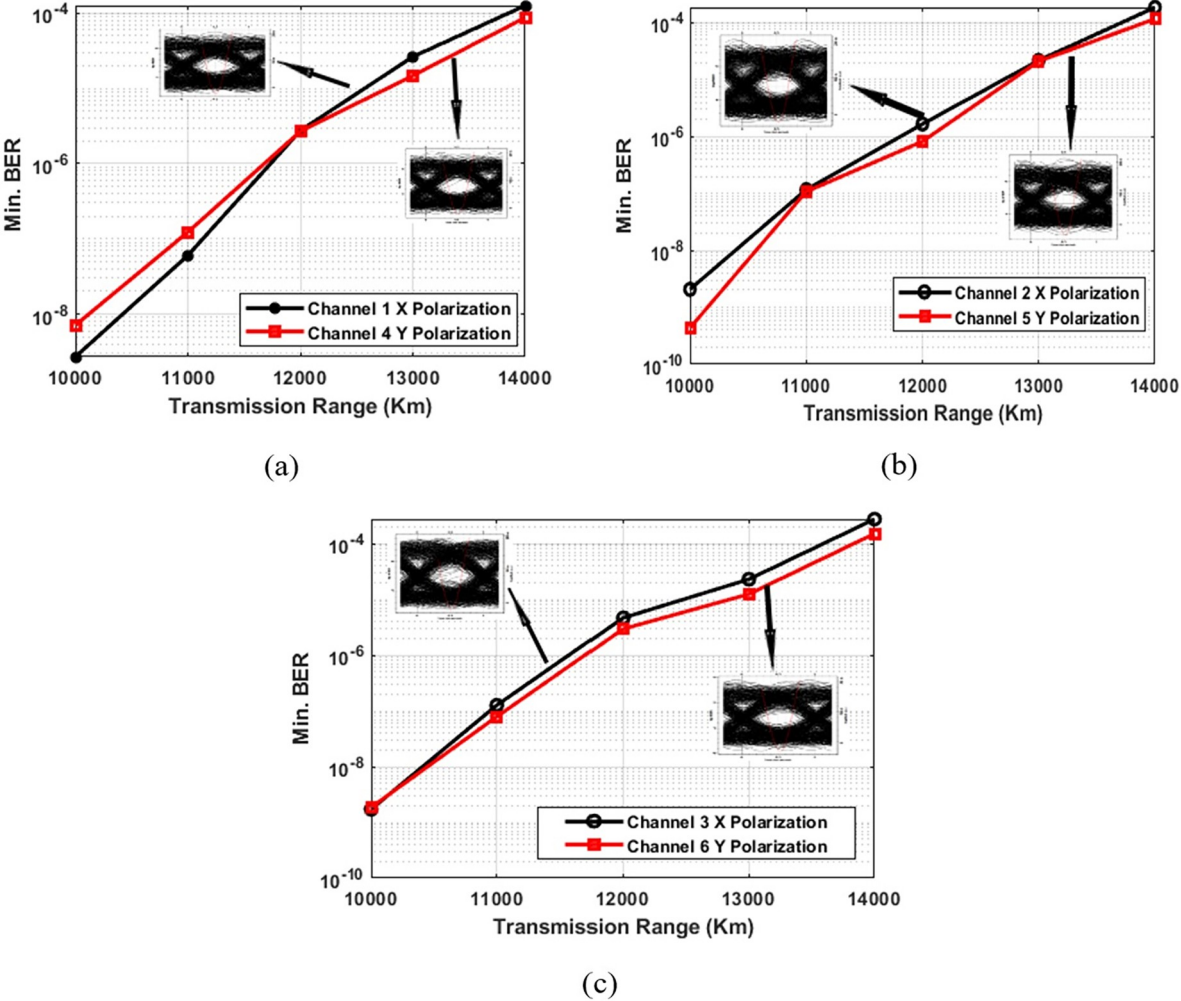
BER performance across varying transmission ranges in the PDM-Is-OWC system (with 4 × 4 MIMO configuration).

To extend the transmission range, a 4 × 4 MIMO system was incorporated. The addition of MIMO significantly extends the transmission range of the Is-OWC system by enhancing spatial diversity and reducing signal fading, leading to improved link performance and increased transmission distances. Fig 4 demonstrates the results achieved with the integrated PDM-MIMO Is-OWC system. The transmission range for all six channels increased from 10,000 km to 14,000 km, with noticeable improvements in BER values at each range. This highlights the success of integrating MIMO with PDM-Is-OWC, leading to an extended range and improved overall link performance. In Fig 4(A), for channels 1 and 4 using MIMO at 14,000 km, Channel 1 (X polarization) has a BER of 9.1 × 10^−03^, and Channel 4 (Y polarization) has 8.7 × 10^−05^. Fig 4(B) shows BER results for channels 2 and 5, where Channel 2 (X polarization) has a BER of 8.1 × 10^−03^ and Channel 5 (Y polarization) shows 9.1 × 10^-03^.Similarly, Fig 4(C) reveals BER results for channels 3 and 6 at 10,000 km. Channel 3 (X polarization) shows a BER of 4.1 × 10^−03^, while Channel 6 (Y polarization) shows 5.1 × 10^−03^. All results are well within the FEC limit, with clear eye openings confirming successful signal reception at 14,000 km.The system’s ability to maintain a BER within FEC limits at 14,000 km shows its potential for deployment in remote sensing applications. Practical implementations could leverage adaptive optics to mitigate pointing errors and real-time CSI for continuous performance optimization.

**Fig 5:**
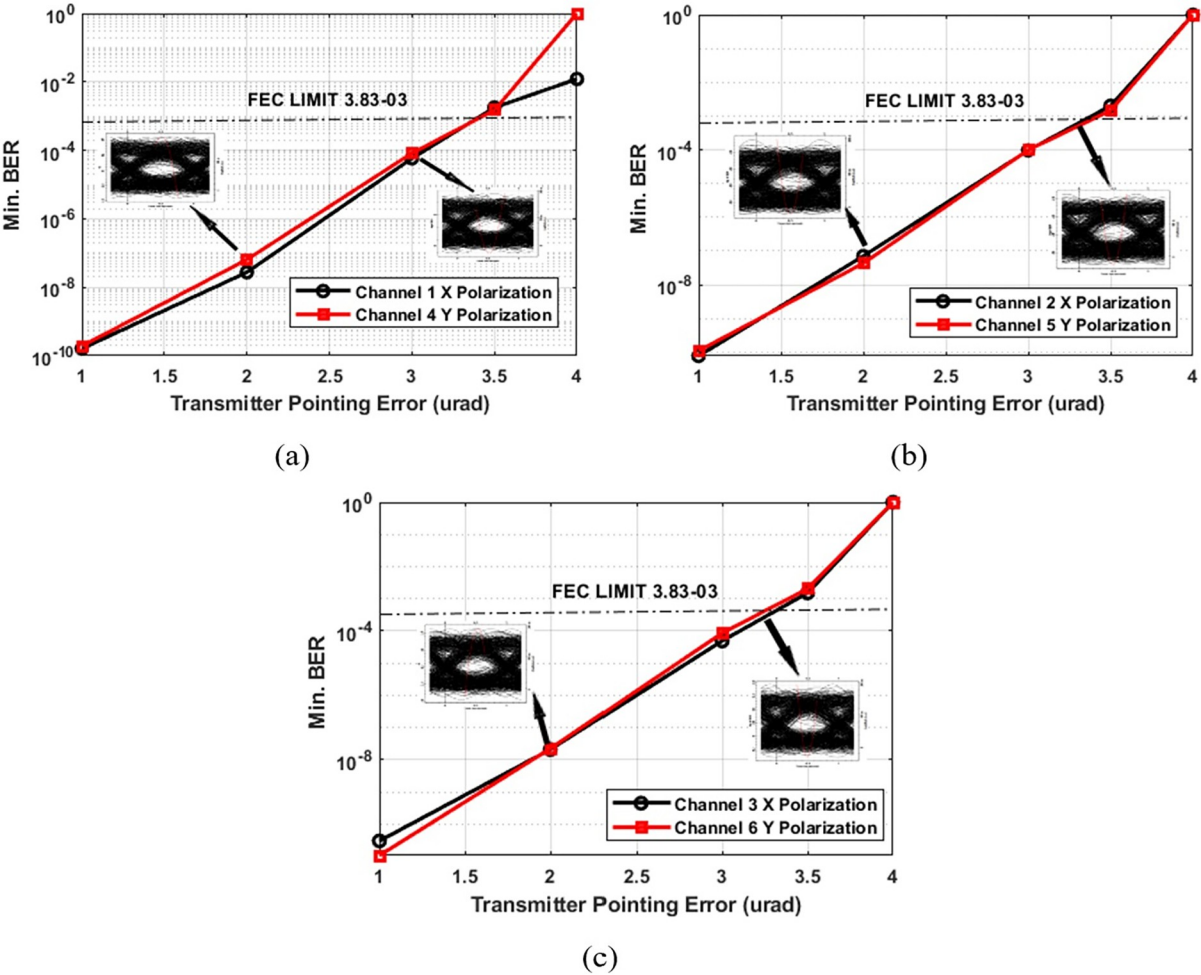
BER performance across varying transmission ranges in the PDM-MIMO-Is-OWC system (with transmitting pointing errors).

**Fig 6:**
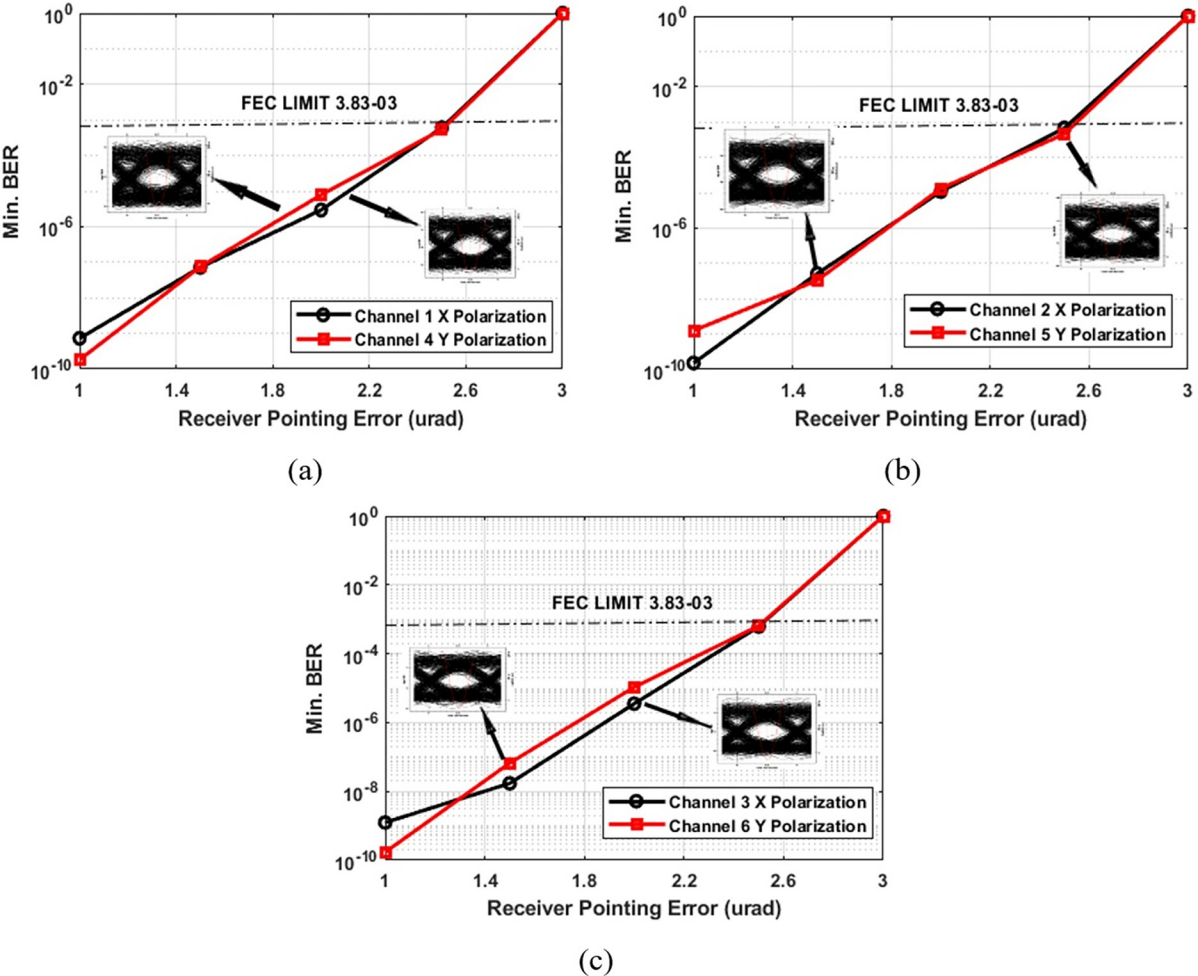
BER performance across varying transmission ranges in the PDM-MIMO-Is-OWC system (with receiving pointing errors).

The Is-OWC system is susceptible to pointing errors, which can significantly impact performance. These errors result from misalignments between the transmitter and receiver, causing signal misdirection and degradation. Minimizing pointing errors is crucial for maintaining stable and reliable optical communication over long distances, especially in space-based applications where precise alignment is essential. To evaluate the impact of pointing errors on the PDM-MIMO Is-OWC system, tests were conducted with pointing errors of up to 4 μrad for the transmitter and up to 3 μrad for the receiver. The transmission range was kept fixed at 6,000 km for simplicity. Fig 5 provides insights into the effects of transmitter pointing errors, while Fig 6 examines receiver pointing errors. These figures offer a comprehensive understanding of how pointing errors influence system performance. In Fig 5(A), for channels 1 and 4, Channel 1 (X polarization) records a BER of 5.8 × 10^−05^, while Channel 4 (Y polarization) shows a BER of 8.23 × 10^−05^. Fig 5(B) presents BER results for channels 2 and 5, where Channel 2 (X polarization) displays a BER of 9.61 × 10^−05^, and Channel 5 (Y polarization) shows a BER of 9.87 × 10^−05^. Fig 5(C) provides insights into channels 3 and 6, where Channel 3 (X polarization) exhibits a BER of 4.90 × 10^−05^, and Channel 6 (Y polarization) demonstrates a BER of 8.57 × 10^−05^. All results are within the FEC limit, even at a transmitter pointing error of 3 μrad, with clear eye openings at a range of 6,000 km.

In Fig 6(A), for channels 1 and 4, Channel 1 (X polarization) records a BER of 6.04 × 10⁻^3^, while Channel 4 (Y polarization) shows a BER of 5.3 × 10^−03^. Fig 6(B) presents BER results for channels 2 and 5, where Channel 2 (X polarization) displays a BER of 6.7 × 10⁻^3^, and Channel 5 (Y polarization) shows 4.6 × 10^−03^. Fig 6(C) provides insights into channels 3 and 6, where Channel 3 (X polarization) exhibits a BER of 6.1 × 10^−03^, and Channel 6 (Y polarization) demonstrates a BER of 6.5 × 10^−03^. Again, all results are within the FEC limit, even at a receiver pointing error of 2.5 μrad. Clear eye openings confirm successful signal reception at a fixed range of 6,000 km.

The results demonstrate that the integration of MIMO and PDM technologies significantly improves the performance of inter-satellite optical wireless communication (Is-OWC) systems. The ability to achieve a reliable data rate of 60 Gbps over a 14,000 km link with acceptable BER values highlights the potential of this system in meeting the growing demand for high-speed data transfer in space-based applications. These results are particularly relevant in the context of satellite networks, where low latency and high throughput are critical. However, the study has certain limitations that must be considered. The simulations assume idealized conditions, which may not fully account for real-world factors like dynamic atmospheric turbulence or more severe pointing errors. While the results provide valuable insights into the system’s capabilities, practical implementations may face additional challenges. Future work could explore more complex atmospheric models and real-time pointing error correction techniques to further improve system resilience. Several factors could influence performance under different operational conditions. For example, stronger atmospheric turbulence or larger misalignments between the transmitter and receiver could lead to higher BER values and degraded system performance. Additionally, longer transmission distances and more significant environmental interference could also affect the results. It is recommended that future studies investigate these variations and implement adaptive techniques, such as FEC and real-time pointing correction, to mitigate these issues. To enhance system performance in practical applications, future work could focus on integrating adaptive optics to compensate for atmospheric disturbances in real time. This could further reduce BER and increase system reliability. Additionally, advanced FEC schemes could be implemented to further improve performance, especially in high-noise environments or under severe pointing error conditions.

## 6. Conclusion

This study presents a novel Is-OWC system that combines PDM and MIMO technologies to achieve high-capacity, long-range communication. The proposed system successfully demonstrates a data rate of 60 Gbps over transmission distances up to 14,000 km with BER values within acceptable FEC limits. These results validate the system’s reliability under challenging conditions, including atmospheric turbulence and pointing errors, highlighting its potential for space-based applications. The integration of PDM improves spectral efficiency by allowing multiple data streams to be transmitted simultaneously, while MIMO enhances link stability through spatial diversity, mitigating interference and signal fading. The robustness of the proposed system suggests it can effectively support high-demand applications such as remote sensing, Earth observation, and satellite-based internet services. Despite its promising performance, the study acknowledges that real-world environmental variations may introduce additional challenges not fully addressed in the current model. Future work will explore the use of adaptive optics and real-time channel state monitoring to further enhance the system’s performance. Additionally, optimization of antenna design and advanced error correction techniques will be investigated to ensure consistent performance in highly dynamic environments. In summary, the proposed PDM-MIMO Is-OWC system offers a practical and scalable solution for high-throughput satellite communication, paving the way for future research and real-world deployment in advanced satellite networks.

## 7. Future work

The proposed PDM-MIMO Is-OWC system has shown significant promise in terms of data throughput, reliability, and long-distance communication capabilities. However, several areas remain open for further exploration to enhance the system’s robustness and adapt it to real-world challenges. One of the key aspects for future research is the integration of real-time MIMO channel state information (CSI). Implementing real-time CSI will allow the system to dynamically adjust to varying atmospheric conditions and pointing errors, which are critical in space-based communication systems where the environment can change rapidly.

Another important area for future development is the optimization of antenna design and configuration. Exploring different antenna arrangements, including varying the spacing and orientation of the antennas, could enhance spatial diversity and reduce signal interference. This will improve the overall system performance, particularly in long-range inter-satellite links where maintaining alignment and minimizing interference are crucial for reliable data transmission. In addition, future work will focus on implementing adaptive optics to correct for atmospheric turbulence in real time. The integration of adaptive optics could significantly improve the system’s resilience to environmental disturbances. Moreover, the use of more advanced FEC techniques could further reduce the BER, even in the presence of severe noise or interference. These enhancements will be key to ensuring the system’s performance in dynamic and challenging environments, such as satellite networks and space-based applications.
